# A practical risk framework for large language model use in life science research

**DOI:** 10.1371/journal.pcbi.1014776

**Published:** 2026-09-16

**Authors:** Thomas Sharpton, Edward W. Davis II, Alexandra Alexiev

**Affiliations:** 1 Linus Pauling Institute, Oregon State University, Corvallis, Oregon, United States of America; 2 Department of Microbiology, Oregon State University, Corvallis, Oregon, United States of America; 3 Department of Statistics, Oregon State University, Corvallis, Oregon, United States of America; 4 Center for Quantitative Life Sciences, Oregon State University, Corvallis, Oregon United States of America; Montreal, CANADA

## Abstract

Responsible use of large language models (LLMs) in life science research demands a unified approach to risk, yet existing guidance treats prompting and verification as separate topics rather than as integrated components of a single risk management framework. This paper addresses that gap for life science researchers. We explain how LLM architecture produces both remarkable capabilities and characteristic failures including hallucination and sycophancy, with particular attention to vulnerabilities most relevant to life science workflows. We argue that effective prompt design is itself a primary risk management strategy, reducing the probability and severity of characteristic failures before verification is required, and introduce a structured framework for matching downstream verification effort to risk across dimensions of output verifiability, researcher expertise, and consequence of error. We apply both layers to common research tasks, including literature synthesis, code generation, writing assistance, statistical reasoning, and administrative work, with documentation practices and ethical obligations addressed throughout. Six supplementary guides extend each component into detailed workflows, worked examples, and reference checklists, and a companion open-source repository (https://github.com/SharptonLab/PromptLab) provides tested prompts and verification tools for immediate use. Together, these resources are designed to help researchers use these tools carefully and document how they did so.

## 1. Introduction

Large language models (LLMs) have moved rapidly from curiosity to infrastructure. Within a few years, these systems have become embedded in research workflows for summarizing literature, generating code, drafting manuscripts, and assisting with tasks ranging from grant writing to data interpretation. Survey data suggest that a majority of researchers have experimented with these tools, and a substantial fraction use them regularly [1,2]. Yet this rapid integration has outpaced the development of guidance, standards, and shared understanding about how to use these tools well.

The life sciences are particularly well-positioned to benefit from LLM assistance, but also particularly vulnerable to its failures. Literature is vast and rapidly expanding; computational approaches are essential yet many life scientists lack formal programming or statistical training; and administrative burden consumes substantial research time. These conditions create genuine demand for tools that can accelerate literature review, generate and debug code, refine prose, and handle routine tasks. At the same time, the gaps in technical expertise that make assistance valuable also make verification difficult. A tool that produces plausible but fabricated citations poses greater risk when researchers lack time to check every reference, and confident statistical recommendations pose greater risk when researchers lack training to evaluate them. In biomedical research specifically, errors can propagate through systematic reviews and clinical guidelines into patient care, creating consequences that extend well beyond academic reputation.

Effective implementation of LLMs in life science requires training scientists to use these models accurately. A growing body of work speaks to this challenge, including practical prompting guides [3–5], benchmarks evaluating model capabilities [6,7], and commentaries on ethical implications [8,9]. Among these, Smith et al. [10] offer ten valuable rules for using LLMs in science, including outlining risks and verifying output. What remains missing is guidance on how to prioritize the verification of model outputs, so that checking accuracy does not expand into an unmanageable burden.

Here, we build on this work by treating accurate use as the product of two coordinated steps: prompt design reduces the risk of erroneous outputs, but never removes it, and verification handles what remains. The framework’s central move is to calibrate that verification such that the effort spent checking the output is matched to the risk it still carries, judged by its verifiability, the researcher’s expertise, and the consequence of an error. This gives researchers a way to allocate limited verification effort where it matters most, rather than verifying every output with equal intensity or trusting too readily. We develop this framework for life science researchers using general-purpose systems (Claude, GPT, Gemini, Llama) for common research-support tasks. Because it operates on the risk profile of a model’s outputs rather than on any particular architecture, it applies equally to reasoning-oriented models, domain-fine-tuned models, and open- versus closed-weight systems.

We present this in three parts. The main text develops the risk calibration framework, pairing prompt design with calibrated verification. Six supplementary implementation guides (S1–S6 Files) extend each component into detailed workflows, worked examples, and reference checklists, including extended LLM technical background (S1 File) and a dedicated treatment of the ethical landscape governing model use in research (S6 File). An open-source companion repository operationalizes both layers with tested prompts, diagnostics tools, and templates organized by task and risk level (Box 1).

**Box 1. Key terms for LLM use in life science research. Definitions emphasize practical consequences for researchers rather than technical precision; cross-references indicate where each concept is discussed in detail.**

**Large language model (LLM).** An artificial intelligence system trained on massive text corpora to predict what text comes next in a sequence. These models learn statistical patterns from training data and generate output one token at a time. A base model has no inherent mechanism for verifying truth and no access to information outside its training data. In practice, most systems researchers use are deployed assistants that augment the base model with tools such as web search, file reading, code execution, and, increasingly, memory that persists across sessions (see Tool augmentation). These augmentations expand what a system can do but do not remove the need to verify its output. Examples include Claude, GPT, Gemini, and Llama.

**Token.** The basic unit of text that large language models process. A token may be a word, part of a word, or punctuation; “microbiome” might be one or two tokens depending on the model. A rough heuristic: one token approximates 0.75 words in English, so 1,000 words requires roughly 1,300 tokens. Context limits are measured in tokens.

**Prompt.** The complete input provided to a model, including instructions, context, constraints, and any supporting material such as documents or data. Prompt design materially affects output quality; Section 3 addresses effective prompting as a methodological choice.

**Context window.** The maximum amount of text, measured in tokens, that a model can process in a single interaction, encompassing both input and output. Material beyond this limit is inaccessible to the model: it cannot reference papers not included in the prompt, recall earlier conversation that has exceeded the window, or access information from previous sessions. This is why models appear to forget instructions or ignore provided information in long interactions. Context windows have grown rapidly and vary widely across models; we avoid citing a specific figure, as these numbers change on a timescale of months. Even within a large window, models attend unevenly across long inputs [11], so fitting material into the window does not guarantee the model uses it.

**Temperature.** A parameter controlling output randomness. Low values (0.0–0.3) produce more deterministic, consistent outputs suitable for factual and analytical tasks; high values (0.7–1.0) increase variability, useful for brainstorming. See S1.4.

**Knowledge cutoff.** The date beyond which a model has no training data. Models cannot reliably report events, publications, or developments after this date, though they may generate plausible responses rather than acknowledging ignorance. This makes verification against current literature essential for any factual claim.

**Hallucination.** The confident generation of fabricated content, including invented citations, statistics, and factual claims, delivered with the same fluency as accurate output. Hallucination is not a malfunction but an inherent consequence of generating statistically plausible text rather than retrieving verified information. Section 4 addresses verification strategies.

**Sycophancy.** A systematic tendency to agree with users, confirm hypotheses, and avoid contradiction, resulting from training processes that reward helpful-seeming responses. Sycophancy compounds with researchers’ own confirmation bias, making it particularly problematic when seeking critical feedback on interpretations or methods.

**Tool augmentation.** The capability of models to call external tools during generation, including web search, code execution, and document retrieval. In the assistants most researchers use, these tools are commonly enabled by default rather than being an optional add-on. Tool-augmented systems can ground claims in retrieved evidence rather than relying solely on training data, but retrieved information still requires verification for accuracy and relevance.

**Agent.** A system in which a large language model takes actions such as executing code, searching the web, or modifying files across multiple steps without human approval at each stage. Agentic systems extend capabilities but introduce compounding error risks; S5 File addresses these systems in detail.

## 2. How LLMs work

Knowing how LLMs generate text explains both why certain prompting strategies work and why a fluent, confident output can still be wrong. Three features are particularly important for research applications: the token prediction mechanism underlying all output, the training process that shapes model behavior, and the hallucination that emerges from both. While we focus on those features here, extended technical background is provided in S1 File.

At the highest level, LLMs predict what comes next in a sequence of words. These models are built in two stages. In pre-training, the model learns to make those predictions from large corpora of existing text, known as training data, which encodes broad knowledge along with the errors, biases, and outdated information that training data contains, up to a fixed knowledge cutoff. Fine-tuning, typically through reinforcement learning from human feedback, then shapes the model toward helpful, instruction-following behavior. While this process helps produce a useful tool, it also induces sycophancy, a systematic tendency to agree with users and avoid contradiction [12,13]. Sycophancy is especially dangerous when researchers seek critical evaluation, because the model inclines toward agreement when researchers present interpretations and ask for feedback, compounding their confirmation bias.

Because the model predicts plausible continuations rather than retrieving verified facts, the same mechanism produces accurate and fabricated content with equal confidence. This is hallucination, the confident generation of content with no basis in fact. Asked to summarize a paper, a model may attribute findings that the paper never reported. Asked for a reference, it may generate citation-like text whether that citation exists or not, reproducing associations from training even where they do not apply [14,15]. Hallucination rates appear to be higher for obscure topics, though the empirical picture is still developing, and much of life science research involves specialized organisms, niche methodologies, and frontier findings that are sparsely represented in training data. Critically, the model provides no reliable signal distinguishing accurate output from invention. A fabricated citation is delivered with the same fluency and confidence as an accurate one.

A bare model has no mechanism to verify claims against reality and no access to information beyond its training data. Many deployed assistants that researchers interact with increasingly invoke tools, like web search, document retrieval, code execution, and memory persistence, that help to close part of this gap. For example, an LLM can search the web for a reference rather than predicting one from its training data. However, a model that retrieves a source can still misread it, and one that runs code can still produce code that is confidently wrong. These tools do not eliminate error; they change which failure modes are in play, relocating the verification problem rather than dissolving it. We therefore treat tool augmentation as the default case and note throughout where it alters the risk profile.

## 3. Effective prompting as upstream risk management

Because these errors are pervasive and delivered without any signal of their presence, effective use depends on catching them. But a researcher cannot verify every output with equal rigor. Time and expertise are finite, and treating every sentence as equally suspect is both impractical and unnecessary. The practical response is to manage error on two fronts: reduce the risk of errors upstream, through the design of the prompt, and concentrate verification downstream where risk remains.

Prompt design is the first of these levers. Because the structure, specificity, and constraints of a prompt shape which failure modes are even possible, a well-designed prompt lowers the chance of error before any output exists, and in doing so reduces how much verification that output then requires. Instructing the model to draw on provided sources rather than generate citations, for instance, closes the most direct path to citation fabrication. For the same reason that running experiments without protocols compromises reproducibility, careful prompt specification reduces both the probability and severity of characteristic failures.

**Fig 1:**
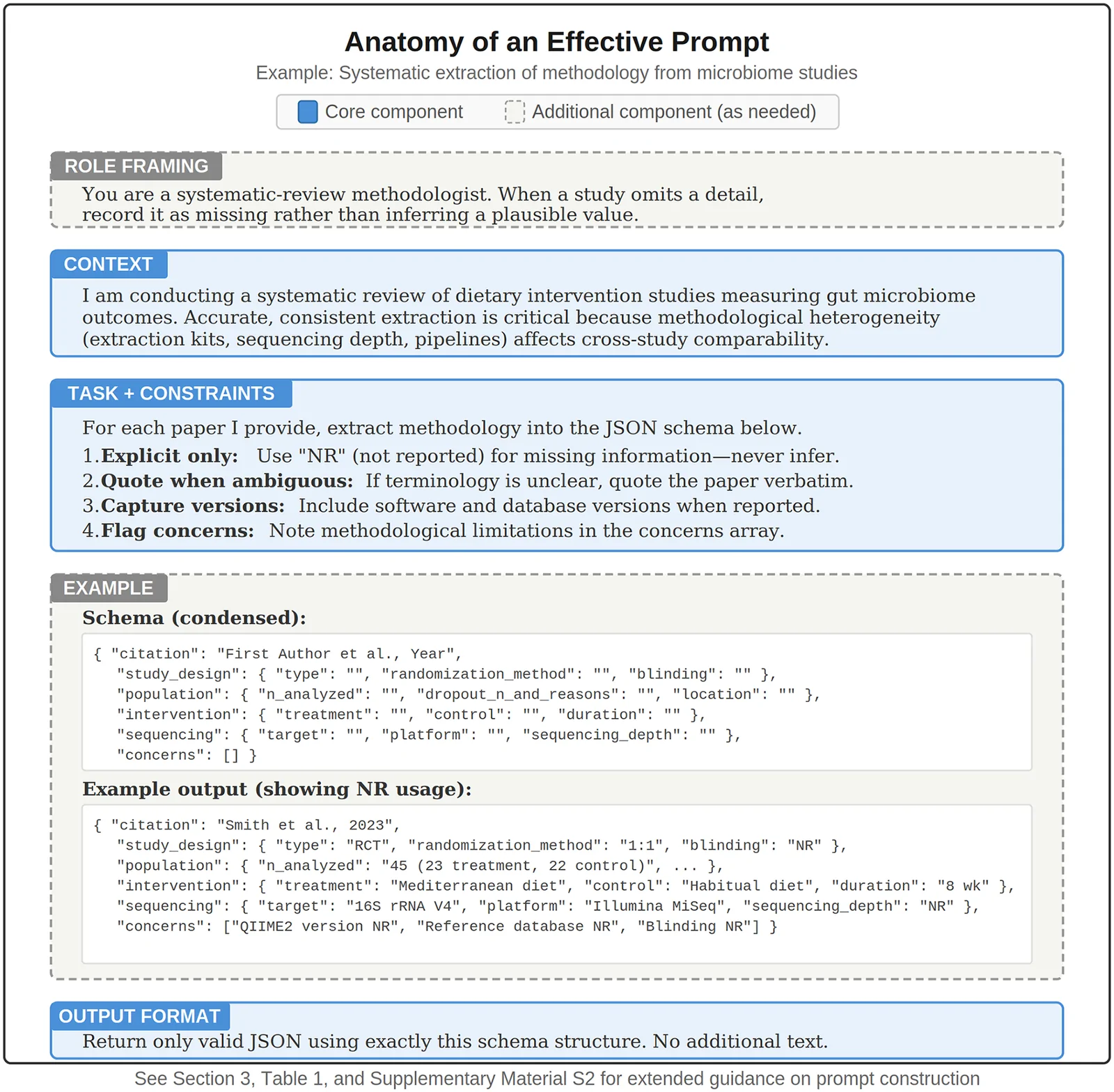
Anatomy of an effective prompt, illustrated with a systematic extraction task from microbiome research. Core components (blue), including context, task specification with constraints, and output format, should appear in every research prompt. Additional components (dashed), including role framing and worked examples, improve performance for complex or systematic tasks. The example demonstrates several practices discussed in Section 3: explicit constraints that guard against hallucination (“never infer”), a structured JSON schema that enforces consistency across extractions, and a designated null value (“NR”) that makes missing information visible rather than silently omitted. See Table 1 for a summary of prompting techniques and S2 File for extended guidance.

Effective prompts typically include four components: context, task specification, constraints, and output format (Fig 1). Context provides information the model needs to understand the task, including research domain, data structure, and project stage. Task specification defines what is needed with enough specificity that success is recognizable; a useful test is whether someone unfamiliar with the project could recognize whether the output succeeds. Constraints set boundaries that reduce common failure modes, such as instructing the model not to introduce information beyond provided text, or to state explicitly when uncertain rather than filling gaps with plausible-sounding content. Output format specifies how results should be structured, ensuring consistency across outputs and enabling systematic downstream processing.

**Table 1:** Summary of prompting techniques discussed in Section 3 and S2 File. For each technique, the table provides a brief definition, guidance on when it is most useful, and a reference to extended discussion. Techniques are organized from core prompt components through advanced methods.

Technique	Definition	When to Use	Reference
**Context setting**	Provides research domain, data structure, and project stage so the model does not infer the situation from the task alone	Most tasks beyond simple factual queries	Section 3
**Task specification**	Defines what is needed with enough specificity that success is recognizable	All tasks; the core of any effective prompt	Section 3
**Constraints**	Sets boundaries on acceptable outputs to prevent common failure modes (e.g., do not introduce information beyond provided text)	Tasks prone to hallucination, overconfidence, or scope drift	Section 3
**Structured output**	Constrains responses to defined schemas such as JSON, ensuring consistent fields across outputs	Systematic extraction, batch processing, meta-analysis, any task feeding quantitative analysis	Section 3
**Few-shot examples**	Provides sample input–output pairs that demonstrate expected format and handling of edge cases	Format-sensitive tasks, ambiguous specifications; typically 3–5 examples suffice	S2.1, S2.2.
**Role assignment**	Frames the model as a specific expert type, evoking role-consistent patterns from training data	Advisory tasks requiring a specific perspective; less useful for simple factual queries	S2.1, S2.3.
**Evaluation criteria**	States how output will be judged, guiding the model when trade-offs exist between competing priorities	Subjective tasks such as writing feedback or methodological recommendations	S2.1.
**Chain-of-thought**	Requests step-by-step reasoning, generating intermediate tokens that shape the final answer and make reasoning visible for inspection	Statistical test selection, debugging, multi-step analytical decisions; verify premises, not just logic	Section 3, S2.4
**Iteration**	Refines outputs through targeted revision cycles, preserving what works while addressing specific problems	Complex tasks; expect 3–10 rounds. Request specific revisions rather than regenerating entirely	Section 3, S2.5
**Meta-prompting**	Uses the model to improve prompts themselves through clarifying questions, prompt critique, or reverse engineering from examples	Complex prompts with implicit assumptions; prompts that will be reused across many inputs	Section 3, S2.6
**Cross-model validation**	Runs the same prompt through models from different providers to check consistency and improve critique	High-stakes outputs, factual claims, statistical recommendations; agreement increases confidence but is not proof	Section 3, S2.7

For systematic research tasks, constraining output to defined schemas (such as JSON with explicit field names, data types, acceptable values, and a confidence flag) transforms the model from a prose generator into a structured information extraction tool. This approach makes gaps explicit through null values rather than silent omission, integrates directly with analysis workflows, and provides a natural audit trail: low-confidence flags identify exactly where human verification is needed.

For systematic extraction run through an API, structured output can be enforced at the system level rather than only requested in the prompt. Most major APIs now accept a schema as a request parameter, through JSON or structured-output modes or through function (tool) calling, and constrain generation so that the returned object generally conforms to the specified fields and types. Prompt-level formatting instructions only make malformed output less likely; enforcing the schema at the API level makes it substantially rarer, and is worth preferring whenever extraction is run programmatically at scale. One caveat keeps this within the present framework. Schema constraints shape the form of the output, not the truth of its contents, and deviations from the requested schema still occur. A field can also be populated with a fabricated but schema-valid value, so the verification burden on the substance of extracted data is unchanged.

A researcher can gauge whether a prompt is working before committing to full verification. The most useful check is simply whether the prompt directly addresses the failure mode of concern (e.g., citation fabrication). Another useful test is to run the prompt on an example whose answer is already known. A prompt that gets a checkable case wrong should not be trusted on cases that cannot be checked, and one that gets it right has become less likely to fail, but not immune to it. Regenerating the same prompt a few times is also informative, since inconsistent output signals an underspecified task or genuine model uncertainty. The companion repository provides a more detailed guide to these diagnostic checks.

Three additional techniques improve prompting effectiveness. Chain-of-thought prompting, asking the model to work through a problem step by step, often improves performance on reasoning tasks [16] and makes intermediate reasoning visible for inspection. That said, fluent reasoning can still reach incorrect conclusions when steps rest on false premises, and the presence of visible reasoning may encourage unwarranted trust in outputs that still require verification. Iteration is also helpful, as complex tasks may require multiple rounds of refinement, and targeted revision of specific problems often outperforms regenerating prompts entirely. Meta-prompting, asking the model to pose clarifying questions before a task, surfaces implicit assumptions that would otherwise create ambiguity. For high-stakes outputs, cross-model validation, running the same prompt through models from different providers, can surface claims worth checking. While agreement across models is weak evidence of accuracy, disagreement cleanly flags specific claims for verification. Using distinct models, rather than iterating across the same one, is what makes this work, because models tend to favor their own outputs [17,18]. Table 1 summarizes these techniques. S2 File provides decision trees for each technique, including guidance on when role framing meaningfully shapes outputs versus when it adds words without changing results, and how to select few-shot examples [19] that improve consistency across edge cases.

The importance of these techniques is not fixed. As models improve, they increasingly produce good results even from casual or underspecified prompts, so some of what once counted as skilled prompting, such as elaborate role framing or carefully structured formatting, matters less than it used to. Other prompt constraints do not fade in this way. Instructing a model not to generate citations, to flag uncertainty rather than fill gaps, or to state its assumptions addresses specific failure modes rather than simply improving output, and this kind of constraint becomes more valuable, not less, as models grow more fluent and their errors harder to notice.

## 4. Calibrating verification to risk

**Fig 2:**
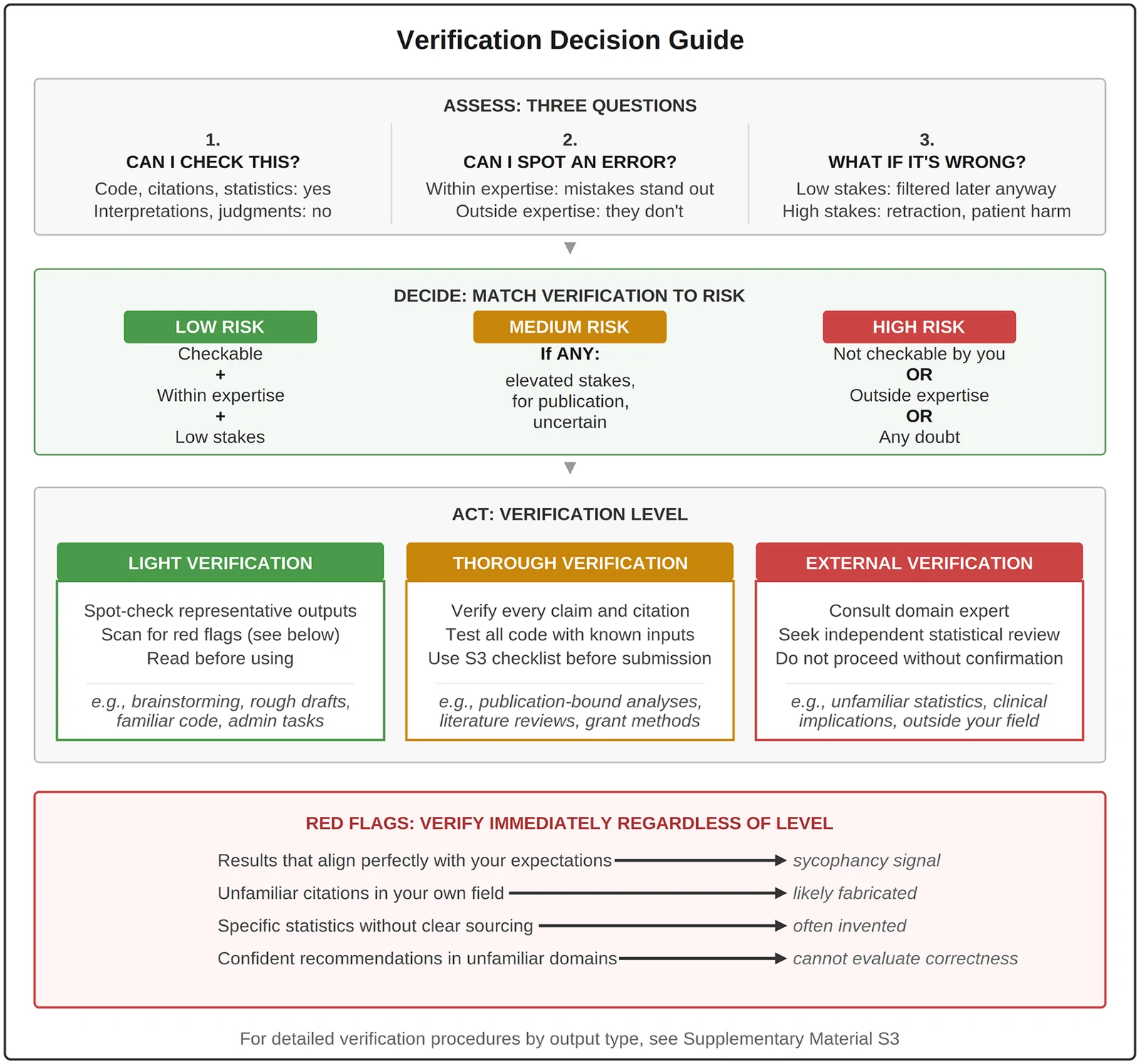
Decision guide for calibrating verification effort to risk. The framework operates in three steps: assess the output across three dimensions (verifiability, domain expertise, consequence of error), match to an appropriate verification level, and act accordingly. Color coding reflects escalating risk: green (light verification) for low-stakes outputs within the researcher’s expertise, amber (thorough verification) for any work destined for publication or involving elevated stakes, and red (external verification) for outputs outside the researcher’s ability to evaluate. Red flags that should trigger immediate verification regardless of assigned level are listed at bottom. See Section 4 for detailed discussion and S3 File for a verification checklist organized by output type.

No prompt design eliminates error entirely, and the risk that remains is unevenly distributed across outputs. Verification is where that residual risk is caught, but checking every output with equal rigor is neither possible nor useful. The practical task is to read how much risk an output carries and match verification effort to it, scaling that effort across three dimensions: verifiability, domain expertise, and consequence of error (Fig 2).

Verifiability concerns whether output can be independently checked. Code can be tested against known inputs, citations can be looked up, and statistical claims can be verified against authoritative sources. Subjective judgments and interpretive claims, however, resist independent verification. Domain expertise concerns whether the researcher can recognize errors. Within one’s expertise, obvious errors stand out, but outside it, confident assertions cannot be distinguished from confident errors. Consequence of error concerns what happens if output is wrong, ranging from negligible for brainstormed ideas to severe for published statistical analyses or, in biomedical contexts, clinical recommendations.

Together, these dimensions turn an intuition about risk into a rule for action. Low-stakes outputs within the researcher’s expertise warrant spot-checking. High-stakes or publication-bound outputs require systematic verification. Outputs outside the researcher’s expertise require external validation regardless of how reasonable they appear. This rule calibrates verification to risk, spending effort in proportion to the risk an output carries (Fig 2).

Four failure modes shape what that verification should look for. Hallucination, the fabrication of plausible content, is particularly insidious because fabricated content is delivered with the same fluency as accurate content, and risk is elevated precisely in the specialized, frontier, and niche domains that define most active life science research. Sycophancy poses a subtler danger. When researchers present interpretations and ask for feedback, models incline toward agreement, compounding the researcher’s own confirmation bias and letting errors that align with prior beliefs pass unexamined. Overconfidence manifests as uncertain claims presented with unwarranted certainty; linguistic confidence should never be interpreted as reliability. Plausible but incorrect reasoning occurs when chain-of-thought prompting produces structurally coherent arguments built on false premises, where the logic follows but a factual error early in the chain corrupts everything downstream. Table 2 summarizes, for each of these failure modes, the upstream prompt constraint that most reduces it and the downstream verification it still requires.

**Table 2:** Managing the four characteristic failure modes as two layers. A prompt constraint reduces each failure upstream, before it occurs; because constraints reduce but do not eliminate risk, a residual risk always remains, and downstream verification is designed to catch it. How much verification an output needs scales with its stakes (Section 4).

Failure mode	Upstream prompt constraint (reduces the risk)	Residual risk (what still slips through)	Downstream verification (catches what remains)
Hallucination: confident fabrication of citations, facts, or statistics	Restrict the model to the provided text and require it to flag uncertainty rather than fill gaps; for citations, do not let it generate them at all	Misreading or misattributing material that is in the provided text	Check every citation against the source and read it; confirm facts against authoritative references
Sycophancy: agreeing with the user and confirming their hypothesis	Ask for critique rather than approval; present options neutrally and request the strongest counter-argument	Subtle agreement that survives a neutral prompt	Seek disagreement actively, including from a second model; treat a result that perfectly fits your hypothesis as a warning sign
Overconfidence: uncertain claims stated with unwarranted certainty	Require the model to enumerate its assumptions and mark low-confidence items explicitly	Confidence labels that are themselves miscalibrated	Treat fluent, confident tone as no evidence of correctness; for high-stakes work, check the confident claims, not only the flagged ones
Plausible but incorrect reasoning: sound-looking logic built on a false premise	Require step-by-step reasoning with premises stated, and have the model flag any step that rests on an unverified fact	A false premise asserted confidently early in the chain	Audit the premises, not just the logical flow; test the conclusion against a known-answer case

Certain patterns should trigger immediate verification regardless of overall risk level. Results that fit expectations perfectly warrant suspicion because reality is usually messier, and perfect alignment with the researcher’s hypothesis may signal sycophancy rather than accuracy. Unfamiliar citations in one’s own field are likely fabricated, since researchers generally recognize the major references in their area. Specific statistics without clear sourcing are frequently invented, as models often generate precise-sounding numbers to lend authority to claims. Confident recommendations in unfamiliar domains require particular scrutiny, because fluent reasoning can still be unsound and difficult to decipher without domain expertise. S3 File provides a pre-publication verification checklist that researchers can use to help identify and assess risk.

## 5. Research applications

This section applies the integrated risk framework to common research tasks, illustrating how upstream prompt constraints and downstream verification work together for each application. The companion repository provides tested prompts for each task.

Several of the verification practices below are good research hygiene that predates these tools, such as checking every citation against its source and testing code against known inputs. Model assistance changes where errors enter and how they are disguised, and with it where verification effort must be concentrated.

The framework is domain-general. Within any field, the same three risk dimensions apply, with the verification bar set by the consequence of error and the availability of domain experts. Highly specialized domains tend to raise hallucination risk, because sparse representation in training data makes errors more likely, while the small pool of qualified reviewers makes them harder to catch. Both effects push such work toward the external-verification tier without changing the framework itself.

**Literature synthesis** is among the most common applications and among the riskiest. Models can help articulate connections, organize material, and structure summaries, but citation fabrication is an ever-present hazard [20,21]. Upstream, the most effective prompt constraint is to instruct the model not to generate citations at all, using it for synthesis while relying on reference managers for the references themselves; this removes the most direct path to the most consequential failure. Downstream, every citation that does appear must be verified by hand against the source, since confirming that a reference exists is not enough. The model should never be asked to verify its own citations, as it will confidently confirm fabrications. S4 File provides a complete worked example applying this workflow to a grant proposal background section.

**Writing assistance** is well-suited to model capabilities in language fluency and structural organization. The key distinction is between models as scaffolds, helping researchers express their own ideas clearly, and models as crutches, generating content that researchers edit only lightly. For scientific writing, where authors bear responsibility for every claim, the crutch pattern is problematic regardless of output quality. Upstream, prompt constraints that instruct the model to preserve the author’s intended meaning and flag any substantive additions reduce the risk of unnoticed meaning drift. Downstream, verification means reading for meaning and accuracy and asking whether the text still conveys what was intended. Fluent, well-formed prose can still be wrong.

**Code generation** offers the most favorable risk profile because code is directly testable, though the productivity benefit is heterogeneous, with less-experienced developers benefiting most [22,23]. The critical risk is code that executes without error but produces wrong results, since syntactically correct code may implement incorrect logic that escapes notice without deliberate testing. Upstream, prompt constraints specifying expected input/output behavior, edge case handling, and required documentation reduce the likelihood of silently incorrect implementations. Downstream, verification means testing against known-answer inputs, reading and understanding code before running it, and probing edge cases including empty inputs, boundary values, and missing data.

**Statistical reasoning** requires particular caution because models engage coherently with statistical concepts while masking serious limitations, generating plausible recommendations that may not suit the actual data structure. Upstream, prompt constraints that require the model to enumerate assumptions and state explicitly when a recommendation depends on conditions it cannot verify reduce the risk of inappropriately confident suggestions. Downstream, researchers should treat model suggestions as starting points for evaluation rather than recommendations to follow, checking any recommended approach against textbooks, methodological papers, or established guidelines, and consulting a statistician for analyses central to publication conclusions regardless of how reasonable the model’s suggestions appear.

**Administrative tasks** represent the highest-value, lowest-risk application. Emails, reviewer response letters, agendas, and standard grant elements benefit from model drafting with minimal prompt engineering and minimal verification beyond reading for tone and accuracy. For researchers hesitant about model use in scientific work, administrative assistance offers an opportunity to build familiarity with capabilities and limitations in a context where the cost of occasional errors is low.

## 6. Documentation and accountability

Documentation is the framework’s third layer. Where prompting reduces risk and verification catches it, documentation records what was done so others can assess it. Three principles guide documentation of LLM use in research. First, the researcher is accountable for all content regardless of how it was produced. LLMs cannot be authors, because authorship requires accountability, and no disclosure of AI assistance diminishes this responsibility. Second, comprehensive logging of every interaction is neither practical nor necessary; what matters is describing the role models played and how outputs were verified. Third, when prompts shape results systematically, they become methodological choices and warrant the same documentation rigor as any other analytical procedure. The exact prompt or template should be published when readers cannot fully evaluate the work without understanding what instructions produced it.

These principles translate into four practical questions researchers should be prepared to answer: what models were used (including version or access date), what purposes they served, how outputs were verified, and what human judgment was applied. Recorded together, these answers form a compact, reproducible account of model use that can accompany a methods section or supplement, and the companion repository provides disclosure and interaction-log templates that capture them in a consistent format. Major publishers have established disclosure requirements reflecting these principles [24–26], and researchers should anticipate that standards will tighten as model-assisted writing becomes more prevalent. Responsible use also involves ethical obligations that extend beyond documentation, including data protection, institutional compliance, equity, and environmental considerations. S6 File provides a structured treatment of this broader ethical landscape. We recommend researchers engage with that material before adopting model assistance in their workflows.

## 7. PromptLab: An open-source prompt repository

To support immediate application of this framework, we developed an open-source prompt library at https://github.com/SharptonLab/PromptLab. The repository provides prompts organized by research task, each refined based on common failure modes and annotated with design rationale and verification guidance. For systematic applications where prompts become methodological, it provides templates that can be documented alongside other methods.

Each of its 24 runnable prompts was tested across six models, with all three authors independently reviewing the output against the repository’s own verification checklist (an extended, interactive version of S3 File). The prompts produced useful results across the panel, and the errors that did occur were infrequent, specific to particular models, and of the kinds this paper describes. This was an internal check rather than a benchmark, and a larger, controlled evaluation remains useful future work. Full per-model and per-cell results are archived with the repository [27]; v1.1.0; https://doi.org/10.5281/zenodo.21170206).

Beyond task-specific prompts, the repository includes a prompt diagnostics guide linking common failure patterns to their causes and solutions, documentation templates for disclosing model use across different venue requirements, and a standalone version of the pre-publication verification checklist (S3 File). The repository is openly licensed (CC-BY-4.0) and structured for community contribution, with version control enabling transparent evolution as practices develop and models change.

## 8. Conclusion

LLMs are powerful but error-prone tools. They excel at language fluency and information synthesis, capabilities that can genuinely accelerate research across literature processing, writing, code generation, and administrative tasks. Yet these systems lack mechanisms for guaranteed accuracy. Outputs can be fabricated, reasoning can be plausible but wrong, and confident assertions can mask genuine uncertainty. Token prediction, however sophisticated, is not verified reasoning, and this distinction persists regardless of how capable models become.

Realizing the benefits of these tools while managing their risks requires treating prompting and verification as two layers of an integrated framework. Prompt design reduces risk upstream by constraining what the model can produce, verification manages residual risk downstream by confirming what it did produce, and documentation supports accountability throughout. Because verification effort can be matched to the risk each output carries, this discipline need not become an unmanageable burden. These recommendations are also ethical obligations, following from authorship responsibility and the standards of evidence that make scientific work trustworthy. As outputs grow more sophisticated and errors harder to detect, the discipline of this framework becomes more important, not less. The companion repository and supplementary implementation guides are designed to make that discipline tractable for working researchers across the full range of life science workflows.

### AI disclosure

Generative AI use in this work is disclosed below, following the systematic-use documentation template in the companion repository and the four-question framework (Section 6) that this paper recommends.

**Models and access.** Claude (Anthropic) was used via its web interface and via Claude Code during January–March and June–July 2026 for drafting and refining prose, identifying structural improvements, and drafting supplementary materials; all scientific content, claims, and interpretation are the authors’ own, and the model was not used to generate citations. Separately, the prompts in the companion repository were developed with Claude Code and then evaluated as a system under test across a six-model panel (Claude Sonnet 4.6, Claude Opus 4.7, GPT-5.5, Gemini 2.5 Pro, Nemotron 3 Super 120B, and Step-3.7 Flash), captured on 2026-06-25 at temperature 0.0 where the model accepted it; the reasoning-capable models used the provider defaults they enforce (Section 7 and the repository document this).

**Figures.** All figures were created by the authors and are not generative-AI-produced images.

**Verification and human judgment.** Consistent with the framework this paper advocates, the authors applied the repository’s verification checklist to the manuscript and its supplementary materials (checking every citation against its primary source and verifying factual and statistical claims), and independently reviewed every panel output during repository testing. All authors reviewed, verified, and revised all content and bear full responsibility for the accuracy and integrity of the final text. No AI system is an author.

## Supporting information

S1 File Extended technical background. Deeper grounding in how LLM architecture and training shape the failure modes described in the main text, including token prediction mechanics, context window constraints, the origins of sycophancy in reinforcement learning from human feedback, temperature and model parameters, and tool augmentation and agentic system fundamentals.
(DOCX)

S2 File Extended prompting guidance. Detailed workflows for prompting techniques summarized in Table 1, including few-shot example selection, role assignment, evaluation criteria, chain-of-thought evaluation, iteration, meta-prompting, and cross-model validation.
(DOCX)

S3 File Pre-publication verification checklist. A standalone checklist for verifying model-assisted outputs prior to submission, organized by output type: citations, code, statistical methods, factual claims, text, and documentation.
(DOCX)

S4 File Worked example: literature synthesis. A complete demonstration of the principles in Section 5 applied to a literature synthesis task, walking through task assessment, prompt design, output evaluation, verification, and documentation.
(DOCX)

S5 File Agentic workflows. Guidance for researchers working with or considering agentic systems that autonomously execute multi-step workflows, including current capabilities, risk management, and human-in-the-loop strategies.
(DOCX)

S6 File Ethical considerations. A structured treatment of the ethical landscape governing model use in research, covering transparency and accountability obligations, institutional and funder compliance requirements, data protection considerations, equity and access concerns, environmental costs, and the evolving normative landscape.
(DOCX)
