# Supplementary material for: A practical risk framework for large language model use in life science research
Source: PLoS Comput Biol. 2026 Sep 16;22(9):e1014776. doi: 10.1371/journal.pcbi.1014776 (PMC13581008; doi:10.1371/journal.pcbi.1014776)
Supplement: Supplementary file 1 [file pcbi.1014776.s001.docx]

# Supplementary Material S1: Extended Technical Background on Large Language Models

This supplementary material extends the technical background presented in Section 2 of the main text. Readers seeking a functional understanding sufficient for applying the framework in the main text need not read further than Section 2; this supplement is intended for researchers who want a deeper grounding in how model architecture and training shape the failure modes described throughout the paper.

## S1.1 Token Prediction and Text Generation

Section 2 of the main text introduces token prediction as the mechanism underlying all large language model output. This section provides additional detail on how that mechanism operates and why its properties matter for research applications.

During training, models process billions of documents and learn statistical patterns: which words follow which, how sentences are structured, what information tends to appear together, and how different writing styles and genres are organized. When generating a response, the model receives an input (the prompt and any preceding conversation) and produces output one token at a time, with each token selected based on patterns learned during training. A token is the basic unit of text that models process. A token may be a word, part of a word, or punctuation; “microbiome” might be one or two tokens depending on the model. A rough heuristic: one token approximates 0.75 words in English, so 1,000 words requires roughly 1,300 tokens. This matters for research applications because context limits, the maximum amount of text a model can process in a single interaction, are measured in tokens.

Because this mechanism generates statistically plausible continuations rather than evaluating truth claims, fluency provides no signal of accuracy. The model has no internal mechanism to distinguish accurate outputs from inventions; both are generated by the same token prediction process and delivered with the same surface confidence. Readers seeking deeper technical understanding of model architectures and training procedures can consult Vaswani et al. (2017), Wolfram (2023), and Shanahan (2024).

## S1.2 The Context Window

The context window is the working memory of a conversation: the literal text the model can reference at any given moment, including the prompt, any preceding conversation, and the model’s own output so far. Unlike knowledge absorbed during training, which is encoded in model parameters and available implicitly, the context window is explicit and finite. Context windows have grown rapidly, vary widely across models, and typically increase on a timescale of months.

This constraint matters because information outside the context window simply does not exist from the model’s perspective. It cannot reference papers not included in the prompt, recall earlier parts of a conversation that have scrolled beyond the window, or access information from previous sessions. Within the context window, models also attend unevenly to different portions of text, with evidence suggesting that material in the middle of very long contexts receives less attention than content at the beginning or end (Liu et al. 2024). Understanding these limitations helps researchers structure prompts effectively, placing critical instructions and constraints at the beginning or end of long prompts rather than burying them in the middle, and interpret why models may appear to ignore provided information in long interactions.

## S1.3 Training, Fine-Tuning, and the Origins of Sycophancy

Section 2 of the main text introduces sycophancy as a characteristic failure mode. This section provides additional detail on how sycophancy arises from the training process and what countermeasures are available.

Pre-training on vast text corpora gives models broad knowledge of topics well-represented in training data, including scientific concepts, methodological conventions, and domain-specific terminology. Yet this same process encodes errors, biases, and outdated information present in those data. Training has a knowledge cutoff date beyond which models have no awareness of events or publications, though they may generate plausible-sounding responses about post-cutoff topics rather than acknowledging ignorance.

Fine-tuning through reinforcement learning from human feedback shapes pre-trained models into helpful assistants, but this process creates sycophancy because human raters tend to score agreeable, helpful-seeming responses more favorably during the training process, and models learn to optimize for these ratings (Sharma et al. 2024; Perez et al. 2023). The result is systematic: models incline toward agreement when researchers present interpretations and ask for feedback, and bias toward yes when asked whether an approach is correct. When a researcher has already formed a view and seeks feedback, a sycophantic model will tend to confirm rather than challenge, allowing errors that align with prior beliefs to pass unexamined. Countermeasures include explicit requests for critique (“identify weaknesses in this argument”), neutral framing that does not telegraph expected answers, and cross-model validation, running the same prompt through models from different providers, which provides a more independent check than same-model self-critique, since models tend to favor their own outputs (Panickssery et al. 2024) and an independent examiner can surface inconsistencies the original model will not volunteer (Cohen et al. 2023). While recent models have made progress reducing sycophancy, the concern remains significant for scientific work where honest critique matters more than validation.

## S1.4 Temperature and Model Parameters

Temperature controls randomness in token selection during generation. Low values (0.0–0.3) produce more deterministic, consistent outputs by concentrating probability on the most likely tokens; these are suitable for factual extraction, code generation, and tasks where consistency across runs matters. High values (0.7–1.0) increase variability by spreading probability more broadly across tokens, which can be useful for brainstorming and creative tasks where diversity of output is valued. For reproducibility-sensitive work, lower temperatures reduce but do not eliminate output variation across runs, since other sources of non-determinism exist in the generation pipeline.

Model selection also matters. Frontier models from different providers have different training data, architectures, and characteristic strengths. When multiple models agree on a factual claim or statistical recommendation, confidence increases, though agreement is not proof of correctness, since models share training data and can make identical errors. When models disagree, the disagreement identifies areas requiring verification rather than indicating which model is correct. Cross-model validation is better suited to high-stakes tasks than routine queries, but for work destined for publication the additional effort is often justified.

## S1.5 Tool Augmentation and Agentic Systems

A bare model generates text from training knowledge and the current context window alone, but most deployed assistants now augment the base model with tools by default, calling external capabilities during generation such as web search, code execution, and document retrieval. Web-connected models can retrieve current information rather than relying solely on training data, partially addressing the knowledge cutoff problem, though retrieved information still requires verification for accuracy and relevance.

Agentic systems go further, enabling models to execute multi-step workflows autonomously: writing and running code, searching and reading documents, and taking actions across multiple steps without human approval at each stage. These capabilities expand what is possible but introduce compounding risks. Errors early in a workflow propagate through subsequent steps, intermediate decisions may not be visible in the final output, and documentation of what an agentic system actually did is substantially more complex than logging a simple prompt-response exchange.

For high-stakes research applications, human checkpoints at consequential decision points remain essential regardless of the apparent capability of the system. Rather than requesting that a system process an entire corpus and return a synthesis, a more prudent approach asks it to process a subset, show extractions for verification, then continue. This maintains meaningful oversight without eliminating the efficiency benefits that motivated using agentic capabilities in the first place. Extended guidance on agentic workflows is provided in Supplementary Material S5.

**References**

Cohen, R., Hamri, M., Geva, M., and Globerson, A. (2023). LM vs LM: Detecting factual errors via cross examination. Proceedings of the 2023 Conference on Empirical Methods in Natural Language Processing (EMNLP 2023), 12621–12640. DOI: 10.18653/v1/2023.emnlp-main.778

Liu, N.F., Lin, K., Hewitt, J., et al. (2024). Lost in the middle: How language models use long contexts. Transactions of the Association for Computational Linguistics, 12, 157–173. DOI: 10.1162/tacl_a_00638

Perez, E., Ringer, S., Lukošiūtė, K., et al. (2023). Discovering language model behaviors with model-written evaluations. Findings of the Association for Computational Linguistics: ACL 2023, 13387–13434. DOI: 10.18653/v1/2023.findings-acl.847

Shanahan, M. (2024). Talking about large language models. Communications of the ACM, 67(2), 68–79. DOI: 10.1145/3624724

Sharma, M., Tong, M., Korbak, T., et al. (2024). Towards understanding sycophancy in language models. International Conference on Learning Representations (ICLR 2024). arXiv: 2310.13548

Vaswani, A., Shazeer, N., Parmar, N., et al. (2017). Attention is all you need. Advances in Neural Information Processing Systems 30 (NeurIPS 2017). arXiv: 1706.03762

Wolfram, S. (2023). What Is ChatGPT Doing … and Why Does It Work? Stephen Wolfram Writings. https://writings.stephenwolfram.com/2023/02/what-is-chatgpt-doing-and-why-does-it-work/
