# Supplementary material for: A practical risk framework for large language model use in life science research
Source: PLoS Comput Biol. 2026 Sep 16;22(9):e1014776. doi: 10.1371/journal.pcbi.1014776 (PMC13581008; doi:10.1371/journal.pcbi.1014776)
Supplement: Supplementary file 2 [file pcbi.1014776.s002.docx]

# Supplementary Material S2: Extended Prompting Guidance

This supplementary material extends the prompting principles presented in Section 3 of the main text. While the main text covers core prompt components (context, task specification, constraints, and output format) and introduces key techniques (chain-of-thought reasoning, iteration, meta-prompting, and cross-model validation), this supplement provides deeper guidance for researchers seeking to develop systematic prompting practices.

## S2.1 Additional Prompt Components

Beyond the four core components described in the main text, complex or systematic work benefits from three additional elements: examples, role framing, and evaluation criteria.

Examples, sometimes called few-shot prompting, communicate expectations more effectively than descriptions for many tasks, particularly when desired output has specific structure or when natural language descriptions would be ambiguous (Brown et al. 2020). Providing sample input-output pairs demonstrates what the researcher wants in a way that definitions cannot fully capture, and models pattern-match against these examples when generating responses. When selecting examples, researchers should choose cases representative of expected variation, including edge cases where errors most commonly occur such as missing data, unusual formats, and ambiguous cases. Examples should be correctly formatted because models replicate errors, and diverse enough to enable generalization while remaining consistent in handling similar situations. A clear structure aids generalization: each example should be explicitly labeled with input and output sections, helping the model identify which parts to pattern-match and which to generate.

Role framing instructs the model to respond as a particular type of expert, drawing on role-consistent patterns from training data. Text following a biostatistician framing differs systematically from text following a science journalist framing because training established what different roles typically produce. Role assignment tends to improve outputs for complex advisory tasks where expertise shapes interpretation: asking the model to act as a biostatistician reviewing experimental design, focusing on power, confounds, and appropriateness of proposed analyses, shapes both what the model attends to and how it frames feedback. Adding behavioral instructions like “be direct about weaknesses” supplements role framing by countering sycophancy that might otherwise soften critical assessment. For simpler tasks including factual queries and routine formatting, role assignment often adds words without meaningfully changing outputs; the model’s default behavior is already appropriate, and the role becomes theatrical rather than functional. Testing with and without role framing reveals whether it makes a difference for any particular task.

Evaluation criteria tell the model how its output will be judged, which shapes priorities when trade-offs exist. Many tasks involve competing considerations: comprehensiveness versus concision, technical precision versus accessibility, speed of response versus thoroughness of analysis. Without explicit criteria, the model balances these according to patterns in training data, which may not reflect researcher priorities. Stating criteria such as “prioritize interpretability over statistical power” or “favor conservative recommendations over novel approaches” provides guidance that affects how the model navigates competing considerations. Evaluation criteria prove particularly valuable for subjective tasks like writing feedback or methodological recommendations, where reasonable outputs could differ substantially depending on what the researcher values most.

## S2.2 Few-Shot Prompting in Practice

While Section S2.1 introduces few-shot prompting, effective implementation requires attention to example selection, structure, and edge cases.

The most common errors in model outputs occur at boundaries: missing data, unusual formats, contradictory information, and ambiguous cases. Including at least one example that demonstrates how to handle such situations prevents the model from improvising solutions that may not match researcher intent. If a researcher is extracting methodology information from papers and some papers lack methods sections, an example showing the desired handling of this case (whether to note “methods not reported,” skip the entry, or flag for manual review) prevents inconsistent treatment across inputs.

Example structure should make the pattern explicit. Labeling sections clearly with markers like “Input:” and “Output:” helps the model identify which parts to match and which to generate. Consistency across examples matters: if the first example uses a particular heading structure or formatting convention, subsequent examples should follow the same pattern. Inconsistency within examples produces inconsistent outputs.

The number of examples involves trade-offs between consistency and context window consumption. For simple format-matching tasks where the pattern is straightforward, two or three examples typically suffice. For complex tasks with substantial variation, more examples may improve coverage but consume tokens that could otherwise provide context or instructions. When context window limits become binding, researchers can sometimes replace multiple similar examples with fewer examples plus explicit instructions describing the pattern, though examples generally communicate more reliably than descriptions alone.

## S2.3 Role Assignment: When It Helps and When It Does Not

Role assignment is widely recommended but inconsistently valuable, and understanding when roles actually improve outputs allows researchers to use this technique strategically rather than reflexively.

Roles help most when the task requires a specific perspective that shapes interpretation, analysis, or communication style. Asking the model to act as a peer reviewer produces different feedback than a generic review request: the peer reviewer framing evokes patterns from training data about what reviewers attend to, how they structure feedback, and what tone they adopt. Similarly, asking the model to explain a concept “as if to a graduate student new to the field” shapes accessibility in ways that generic instructions about clarity do not fully capture. The role provides implicit context about audience, purpose, and conventions that would otherwise require explicit specification.

Roles help less for tasks where the model’s default behavior is already appropriate. Asking the model to “act as an expert” when answering a factual question about a well-documented topic typically produces the same response as asking the question directly: the model already draws on expert-level text from training when generating responses about technical topics. Testing reveals the difference. Running a prompt with and without role framing shows whether results differ meaningfully; if outputs are essentially equivalent, role assignment can be removed without loss.

When using roles, specificity matters. “Act as a biostatistician specializing in longitudinal study design” evokes more targeted patterns than “act as a statistician,” which evokes more targeted patterns than “act as an expert.” The more specifically the role matches the actual expertise needed, the more likely role framing is to improve outputs. Combining roles with explicit behavioral instructions often works better than roles alone: roles establish perspective but may not override default behaviors like sycophancy, so instructions like “be direct about weaknesses” or “identify problems even if they seem minor” supplement the role with explicit behavioral guidance.

## S2.4 Chain-of-Thought Reasoning: Benefits, Risks, and Evaluation

The main text introduces chain-of-thought prompting as a technique that improves performance on reasoning tasks by generating intermediate steps (Wei et al. 2022). This section provides guidance on when to use chain-of-thought, how to evaluate the reasoning produced, and how to mitigate the risk of false confidence.

Chain-of-thought proves most valuable for tasks with clear logical structure where intermediate steps can be evaluated. Statistical test selection benefits because the reasoning should address data structure, assumptions, and fit; each step can be checked against methodological knowledge. Code debugging benefits because reasoning should identify the error, explain why it occurs, and justify the fix; each step can be verified against the actual code behavior. Literature synthesis benefits less because the reasoning (“this paper relates to that paper because…”) is harder to evaluate systematically and may simply rationalize associations the model would have made regardless.

Evaluating chain-of-thought outputs requires checking both logical connections and factual content. A common error is verifying that each step follows from the previous without verifying that the factual claims within steps are accurate. A statistical recommendation might present coherent reasoning (“the data are paired, so a paired test is appropriate; the distribution appears non-normal, so a non-parametric test is preferred; therefore, use the Wilcoxon signed-rank test”) where the logic is valid but the premise is wrong (perhaps the data are not actually paired, or the sample size is large enough that normality assumptions matter less). Researchers should explicitly check factual claims within reasoning against their understanding of the actual situation rather than only following the logical flow.

When chain-of-thought produces conclusions that seem wrong despite coherent reasoning, several diagnostic questions help identify the problem. Does the reasoning rest on factual claims about the specific situation that should be verified? Does the reasoning make assumptions that were not stated in the prompt and may not hold? Does the reasoning apply general principles correctly to the specific case? Often, prompting the model to reconsider specific steps (“Are you certain the data are paired? The study design involves different subjects in each condition.”) reveals where reasoning went wrong.

## S2.5 Iteration: Patterns, Signals, and Recovery

The main text establishes that iteration is expected practice for complex tasks, with three to ten rounds typical. This section provides guidance on effective iteration patterns, signals that iteration is not converging, and strategies for recovery when prompts reach local optima.

Effective iteration preserves what works while targeting what does not. Requesting specific revisions (“simplify the third paragraph while keeping the technical content” or “the structure is good but examples are too generic—replace with examples from microbiome research”) focuses the model on actual problems rather than regenerating satisfactory portions. Building complex outputs incrementally also improves results: generating an outline first, refining the outline, then expanding sections allows evaluation at each stage before investing in full development. This approach also makes recovery easier when problems emerge, since restarting from a working outline costs less than restarting from nothing.

Several signals indicate that iteration is not converging productively. Minimal change across rounds, where three or four iterations produce outputs that differ only superficially, suggests the prompt may be underconstrained (allowing multiple acceptable solutions) or overconstrained (preventing the model from achieving what’s requested). Return to previous formulations, where the model reverts to phrasings or structures from earlier rounds despite requests to change them, suggests the requested changes may conflict with strong patterns in training data. Attention to surface features while missing underlying issues, where revisions address word choice or sentence structure but not logical gaps or substantive problems, suggests the model may not have understood what actually needs to change.

When iteration stalls, reconsidering the prompt structure is often more productive than continued refinement. Questions worth asking include whether the task specification actually captures the goal, whether constraints are preventing desired outputs (sometimes constraints conflict with each other or with implicit expectations), whether different examples would demonstrate expectations more clearly, and whether the task should be decomposed into subtasks that can be addressed sequentially. Starting fresh with a restructured approach is often faster than continuing to refine something fundamentally misaligned.

## S2.6 Meta-Prompting: Using Models to Improve Prompts

The main text introduces meta-prompting as a technique for surfacing implicit assumptions before task execution. This section describes three meta-prompting patterns in detail: the interview method, prompt critique, and reverse engineering from examples.

The interview method asks the model to pose clarifying questions before providing the task. A researcher might say “I want help with a task, but before I describe it fully, ask me questions that would help you do it well. The task involves summarizing papers for a literature review.” The model typically asks about target audience, desired length, how to handle contradictory findings, what to do with methodological limitations, and similar considerations. These questions force researchers to articulate requirements they had not explicitly considered, often revealing assumptions that would otherwise create ambiguity. The resulting prompt, informed by answers to these questions, is typically more precise than what researchers write unaided. This approach adds one round-trip to the interaction but pays dividends for complex tasks where implicit assumptions are likely to cause problems.

Prompt critique asks the model to evaluate a draft prompt before deployment. A researcher might present a prompt planned for extracting methodology details from papers and ask “What is ambiguous in this prompt? What assumptions might not hold for all papers? How could I improve consistency across extractions?” Models often identify ambiguous terminology that could be interpreted multiple ways, unaddressed edge cases such as papers without methods sections, missing constraints that would prevent common errors, and opportunities for clearer structure. This approach is particularly valuable for prompts that will be reused many times, where investing in prompt quality pays off across multiple uses.

Reverse engineering from examples works when the desired output is clear but hard to specify. A researcher might show three examples of good methodology summaries and ask “What prompt would reliably produce summaries like these?” The model infers implicit criteria from the examples and makes them explicit, often identifying patterns the researcher followed intuitively but did not articulate. This approach is particularly valuable when desired output involves subtle stylistic or structural choices that are easier to demonstrate than describe.

## S2.7 Cross-Model Validation: Implementation Details

The main text introduces cross-model validation as a strategy for improving reliability, particularly for high-stakes outputs. This section provides implementation details, guidance on when cross-validation adds value, and interpretation of agreement and disagreement.

Cross-validation provides most value in four situations. When factual claims matter, different training data means agreement across models increases confidence that claims are accurate. When statistical or methodological recommendations are involved, different models may have different biases or draw on different sources, making consensus more meaningful. When code will be used in analyses or publications, different implementation choices across models can reveal robust versus fragile solutions. When stakes are high and single-model outputs seem uncertain, multiple perspectives reduce the risk of undetected errors.

The overhead of cross-validation is not warranted for routine tasks with easily verified outputs, stakes low enough that occasional errors are acceptable, time constraints that preclude additional processing, or tasks where researcher expertise suffices for direct evaluation. For simple factual queries or routine formatting, verification through expertise or quick reference checking is more efficient than running multiple models.

For implementation, running the same prompt through two or three models from different providers (Claude, GPT, Gemini) provides useful diversity. Agreement increases confidence but is not proof of correctness; models share training data and can make identical errors, particularly for common misconceptions or widely-repeated inaccuracies. Disagreement does not indicate which model is correct but rather that the question may lack a clear answer, that models have different information or different biases, or that the prompt is ambiguous in ways that different models resolve differently.

Cross-validation proves particularly valuable for critique tasks. A single model tends toward sycophancy with its own outputs, producing mild, hedged criticism when asked to evaluate what it just generated. Asking a different model to critique the output tends to produce more useful feedback than same-model self-critique, since models exhibit self-preference bias toward their own outputs (Panickssery et al. 2024) and an independent examiner can surface inconsistencies the original model will not volunteer (Cohen et al. 2023). For important documents or analyses, generating with one model and critiquing with another surfaces problems that same-model critique would understate.

## S2.8 Matching Complexity to Task

Not every prompt needs every component, and over-engineering prompts for simple tasks wastes effort while under-engineering prompts for complex tasks produces unreliable results. Matching prompt complexity to task complexity is the guiding principle.

Simple tasks warrant simple prompts. Factual queries within researcher expertise, where answers can be verified immediately, need only the question itself. Routine formatting tasks with clear specifications need only the format description and input. Adding context, examples, and elaborate constraints to such tasks consumes time without improving results.

Complex tasks warrant full scaffolding. Tasks where models must make judgment calls that will inform decisions benefit from context establishing the situation, constraints preventing likely failure modes, examples demonstrating expectations, and evaluation criteria specifying priorities. Statistical recommendations, literature synthesis, and methodological advice all fall into this category. For such tasks, time invested in prompt development typically saves time in iteration and verification.

The test for appropriate complexity is whether the researcher can justify why each component is or is not included. If a component would not change the output, it probably is not needed. If the output could be wrong in ways a component would prevent, the component is probably warranted. The more complex the task, the higher the stakes of errors, and the more desired output differs from what the model would produce by default, the more prompt structure is justified.

## S2.9 Structured Outputs at the API Level

The main text (Section 3) notes that constraining output to a defined schema turns a model into a structured extraction tool, and that for programmatic extraction this constraint can be enforced at the system level rather than merely requested in the prompt. This section develops that point for researchers running extraction at scale.

When a model is accessed through an API rather than a chat interface, most major providers accept a schema as a request parameter. Two related mechanisms are common. In the first, sometimes called structured-output or JSON mode, you give the model a template that lists the fields you want, the type of value each should hold, and which are required; the system then forces every response to match that template. The second, called function or tool calling, works the same way but frames the task as filling in the inputs to a function you have defined. In both cases the benefit is the same: the response returns in the exact structure requested, with the right fields present, so it can be fed straight into an analysis without the cleanup and error-handling that prompt-based formatting usually requires.

This reliability changes the downstream workflow. Prompt-level formatting requires defensive parsing and retry logic to handle the cases where the model adds commentary, omits a field, or returns invalid JSON; schema enforcement removes that burden, which matters when extraction runs across hundreds or thousands of records. It also makes missing information explicit through null or designated placeholder values rather than silent omission, and it pairs naturally with a per-record confidence field that flags where human review is needed.

One caveat keeps this within the framework of this paper. Schema enforcement guarantees the form of the output, not the truth of its contents. A required field will be populated, but it can be populated with a fabricated yet perfectly schema-valid value, and a confidence flag is itself a model output that can be miscalibrated. Structured outputs therefore reduce formatting and completeness failures rather than hallucination; the verification burden on the substance of extracted data is unchanged, and systematic extraction still requires the spot-checking and source verification described in Section 4 and Supplementary Material S3.
