# Supplementary material for: A practical risk framework for large language model use in life science research
Source: PLoS Comput Biol. 2026 Sep 16;22(9):e1014776. doi: 10.1371/journal.pcbi.1014776 (PMC13581008; doi:10.1371/journal.pcbi.1014776)
Supplement: Supplementary file 3 [file pcbi.1014776.s003.docx]

# Supplementary Material S3: Pre-Publication Verification Checklist

This supplementary material provides a structured checklist for verifying work that involved large language model assistance before submission for publication, grant review, or other high-stakes contexts. The checklist operationalizes the verification framework presented in Section 4 of the main text, organizing verification tasks by output type. Not every section applies to every project; researchers should focus on sections relevant to their specific use of model assistance.

## S3.1 Citations and References

Citation fabrication represents one of the most common and consequential failure modes of large language models. Models routinely generate citations complete with plausible author names, journal titles, and findings, delivered with the same confidence as accurate references. Researchers should assume all model-suggested citations are fabricated until independently verified.

Before submission, researchers should confirm that:

- Every citation has been verified to exist through PubMed, Google Scholar, or the appropriate disciplinary database
- Each cited paper has been located and its content reviewed
- Claims attributed to sources accurately reflect what those sources actually say
- Bibliographic details including authors, year, journal, volume, and pages are correct
- No citations generated by the model were accepted without independent verification

The model should never be asked to verify its own citations, as it will confidently confirm fabricated references.

## S3.2 Code and Computational Methods

Code that executes without errors may nonetheless produce incorrect results. Syntactically valid code can implement flawed logic, mishandle edge cases, or produce subtle errors that escape notice without systematic testing.

Before submission, researchers should confirm that:

- All generated code has been read and understood, not merely executed
- Code has been tested with known-answer inputs where correct outputs can be independently determined
- Edge cases including empty inputs, boundary values, and missing data have been tested
- Packages and functions used are current and not deprecated
- Logic is appropriate for the actual data structure being analyzed
- Results have been spot-checked against manual calculations or alternative implementations

## S3.3 Statistical Methods and Recommendations

Large language models can engage with statistical concepts in ways that sound authoritative while masking serious limitations. Recommendations may seem reasonable but violate assumptions given the actual data structure, or may omit critical caveats on which validity depends. Model suggestions should be treated as brainstorming rather than authoritative guidance.

Before submission, researchers should confirm that:

- Any model-suggested statistical approach has been verified against textbooks, methodological papers, or established guidelines
- Assumptions required by the recommended approach have been checked against the actual data
- For analyses central to the work’s conclusions, a statistician or methodologist has reviewed the approach
- Alternative approaches have been considered rather than accepting the first suggestion

## S3.4 Factual Claims

Confidently stated false claims are indistinguishable from accurate ones without independent verification. Models generate precise-sounding numbers and specific details that lend authority to claims regardless of whether those details are accurate.

Before submission, researchers should confirm that:

- Specific numbers, percentages, and statistics have been traced to verifiable primary sources
- Claims about mechanisms, methods, or established findings have been verified against primary literature or authoritative references
- Historical or contextual claims have been checked for accuracy
- Any claim that would be notable if true has been independently verified

## S3.5 Text and Writing Assistance

Model editing can subtly shift meaning, introduce unsupported claims, or distort emphasis in ways that may escape notice when reviewing only for grammar and flow. Text should be reviewed for meaning and accuracy, since fluent prose can still be wrong.

Before submission, researchers should confirm that:

- All text has been reviewed for meaning and accuracy, not only grammar and flow
- No unsupported claims were introduced during drafting or editing
- Technical terminology is used correctly throughout
- The final text accurately represents the data, methods, and conclusions
- The researcher’s intended meaning has been preserved through any model-assisted revision

## S3.6 Documentation

Adequate documentation supports reproducibility and demonstrates the due diligence expected of authors who bear full responsibility for all content.

Before submission, researchers should confirm that:

- Models used are recorded, including name and version or access date where available
- The nature of model assistance is documented appropriately per target venue requirements, whether in Methods, Acknowledgments, or Author Contribution statements
- Prompts used for systematic work that shaped results are preserved and available for disclosure
- Verification procedures undertaken have been documented

## S3.7 Final Verification

The principle underlying this checklist is that researchers bear responsibility for every claim in their work, regardless of how that claim was generated. Model assistance does not reduce this responsibility; it may increase it by introducing errors the researcher did not make independently.

Before submission, researchers should be able to affirm that:

- No model output was accepted without verification appropriate to its stakes
- The researcher is prepared to defend every statement in the work, because authorship entails accountability for all content regardless of its origin
