# Supplementary material for: A practical risk framework for large language model use in life science research
Source: PLoS Comput Biol. 2026 Sep 16;22(9):e1014776. doi: 10.1371/journal.pcbi.1014776 (PMC13581008; doi:10.1371/journal.pcbi.1014776)
Supplement: Supplementary file 4 [file pcbi.1014776.s004.docx]

# Supplementary Material S4: Worked Example

This supplementary material demonstrates the guidance from the main text applied to a realistic research task: synthesizing literature on the gut-brain axis in animal models. The example walks through task assessment, prompt design, verification, and documentation.

## S4.1 The Task and Its Assessment

A researcher is preparing the background section for an R21 grant proposal on gut microbiome influences on anxiety-like behavior. The task requires synthesizing recent literature on gut-brain axis mechanisms in rodent models, identifying established findings, methodological approaches, and gaps that justify the proposed research. Before beginning, the researcher must assess whether this task is appropriate for model assistance and what verification it will require.

Several features make this task suitable for model assistance. Synthesis across multiple papers plays to model strengths in pattern recognition and information organization. The researcher has domain expertise sufficient to evaluate outputs for accuracy. The output is a draft requiring revision rather than a final product. Time pressure makes acceleration valuable. Despite these favorable features, this task carries substantial risk. Literature tasks have high hallucination risk, particularly for citations (Walters and Wilder 2023; Buchanan et al. 2024). Grant proposals are high-stakes documents where errors damage credibility. Synthesis claims must accurately represent the underlying literature. Fabricated citations discovered by reviewers would undermine the entire proposal.

Applying the verification framework from Section 4, the researcher considers three dimensions. Verifiability is mixed: synthesis claims can be verified against source papers and citations can be checked in databases, but completeness of coverage is harder to verify. Domain expertise is high: the researcher works in this field and can recognize whether claims align with established understanding. Consequence of error is high: grant proposals are reviewed by experts who will recognize fabricated citations or misrepresented findings. This assessment indicates systematic verification is required. Every citation must be verified. Synthesis claims must be checked against source papers. The output is a starting point, not a finished product.

## S4.2 Prompt Design

The researcher designs a prompt incorporating constraints that prevent predictable failure modes.

An initial, problematic prompt might simply ask the model to summarize recent literature on gut-brain axis and anxiety in rodent models for a grant proposal. This prompt is insufficient for several reasons: it provides no context about the researcher’s specific focus or existing expertise, it includes no constraints preventing hallucination, it specifies no output format, it provides no guidance on handling uncertainty, and it implicitly invites the model to generate citations, which carries high hallucination risk.

A revised prompt addresses these shortcomings. The researcher specifies that they are writing the background section for an R21 proposal on how gut microbiome composition influences anxiety-like behavior in mice, that the proposed work will use germ-free and gnotobiotic mouse models to test specific bacterial metabolites, and that they need to establish what is known about gut-brain axis mechanisms relevant to anxiety. The prompt then requests a structured overview based on training data, covering established mechanisms linking gut microbiota to brain function and behavior, key methodological approaches used in the field including germ-free models, antibiotic treatment, and probiotic interventions, and what remains unclear or contested that would justify new research. The prompt instructs the model not to provide specific citations, noting that the researcher will find and verify citations independently using the overview as a guide. The prompt further instructs the model to clearly distinguish between well-established findings with multiple replications and broad consensus versus preliminary findings from single studies or contested interpretations, to state explicitly when uncertain whether something is well-established, and not to fabricate specific experimental details, statistics, or researcher names. Finally, the prompt specifies organization with clear headers for each section, indication of whether each major claim is well-established or preliminary, and a concluding section identifying open questions.

The prompt design reflects several principles from Section 3. Context establishes the specific application, model system, and focus, helping the model provide relevant rather than generic information. Task specification requests a structured overview with specific deliverables. Constraints are crucial: prohibiting citations prevents the highest-risk failure mode, and requiring distinction between established versus preliminary findings counters overconfidence. Output format makes verification tractable. The closing note that accuracy of the conceptual landscape matters more than comprehensiveness reduces pressure to fabricate.

## S4.3 Model Output and Evaluation

The model produces a response organized as requested. On established mechanisms, the model describes vagal signaling as well-established, noting that the vagus nerve provides direct communication between gut and brain and that studies indicate some microbiota-mediated effects require intact vagal signaling. Microbial metabolites are described as well-established with active investigation, noting that short-chain fatty acids can influence brain function while specific pathway details remain under investigation. Immune signaling is described as well-established. Tryptophan metabolism is described as established but complex, with the model noting uncertainty about current consensus on how directly gut bacterial tryptophan metabolism affects brain serotonin.

On methodological approaches, the model notes that germ-free mice consistently show altered anxiety-like behavior compared to conventional controls, though the direction of effect varies across studies and behavioral tests. The model characterizes this variability as itself an established finding in the literature.

On gaps and open questions, the model identifies mechanism specificity (identifying which specific bacteria and metabolites are responsible remains challenging), translation to behavior (the gap between molecular mechanisms and complex behaviors remains substantial), and individual variation (why manipulations produce variable effects across studies is not well resolved).

The researcher first evaluates the output against domain expertise. Vagal signaling as an established pathway is consistent with the researcher’s understanding. Short-chain fatty acids as established with ongoing mechanism work is accurate. Uncertainty about the tryptophan-serotonin relationship is appropriately hedged, as this is genuinely contested. Variable germ-free anxiety phenotypes captures an important nuance. The gaps identified are real. At this stage, the conceptual structure appears sound with no obvious hallucinations at the claim level.

## S4.4 Verification Workflow

Because the prompt instructed no citations, the researcher conducts an independent literature search to verify that claims are supported, to find specific citations for the grant proposal, and to identify findings the model may have missed.

The search strategy involves PubMed search for gut-brain axis, anxiety, and mouse, filtered first to reviews then to primary literature. The researcher follows citations from known key papers and checks for recent papers beyond the model’s training data cutoff.

The outcome is that supporting literature is found for all major claims. Variability in germ-free phenotypes is confirmed by multiple reviews. Tryptophan-serotonin uncertainty is validated, with genuine disagreement in the literature. Two recent papers published after the training cutoff strengthen the case for the proposed work.

The researcher then checks for omissions and identifies limited discussion of sex differences (an important methodological consideration), no mention of the hypothalamic-pituitary-adrenal axis (relevant to stress and anxiety), and recent work on specific bacterial species not mentioned in detail. Assessment: omissions are understandable given the prompt’s mechanism focus. The researcher will add these elements during revision.

## S4.5 Integration and Final Documentation

The researcher uses the verified output as structural scaffold rather than finished draft. The model output provides organizational structure, appropriate framing of established versus emerging findings, and language for characterizing gaps. The researcher adds specific citations from independent search, recent findings beyond training data, missing topics (sex differences, HPA axis), connection to specific aims, and their own scholarly voice. The final text is substantially rewritten, using the model output as conceptual scaffolding rather than prose to be lightly edited.

Following Section 6 of the main text, the researcher is prepared to answer the core disclosure questions. What models were used: the specific model and version, recorded along with the access date. What purposes they served: generated structured overview of gut-brain axis literature as guide for background section, with prompt explicitly instructing no citations. How outputs were verified: conceptual structure verified against domain expertise, all citations from independent literature search (PubMed, Google Scholar) and verified against original sources. What human judgment was applied: final text substantially written by author, with interpretation, emphasis, and connection to specific aims representing the author’s contribution.

This preparation supports disclosure in whatever format the target journal or funder requires.

## S4.6 What Could Have Gone Wrong

Several alternative approaches would have produced worse outcomes. With naive prompting asking for a literature review without constraints, the model would likely generate plausible citations that may not exist, present claims without uncertainty markers, and produce output accepted with light editing. A fabricated citation discovered by reviewers would destroy proposal credibility.

With overreliance on output, using model text as near-final with quick citation additions, the voice sounds generic, important recent papers are missed, nuances are lost, and gaps do not connect to specific aims. The proposal reads as AI-assisted in ways that undermine rather than enhance credibility.

With skipped verification, thoughtful prompt design but no verification because output appears correct, conceptual errors may be present, overconfident claims may misrepresent contested findings, and no documentation supports disclosure if questioned.

## S4.7 Generalizing the Workflow

This workflow generalizes across literature tasks and, with appropriate modifications, to other applications. The core sequence involves assessing verification needs before starting, designing prompts that prevent predictable failures, requesting uncertainty markers, verifying systematically using expertise and independent sources, using output as scaffold rather than final product, and being prepared to answer disclosure questions.

Specific constraints differ by task. Code requires testing rather than citation avoidance. Statistics requires expert consultation rather than literature search. Administrative tasks require lighter verification. But the underlying workflow applies throughout: assess risk, constrain the prompt, verify the output, document the process.
