# Supplementary material for: A practical risk framework for large language model use in life science research
Source: PLoS Comput Biol. 2026 Sep 16;22(9):e1014776. doi: 10.1371/journal.pcbi.1014776 (PMC13581008; doi:10.1371/journal.pcbi.1014776)
Supplement: Supplementary file 5 [file pcbi.1014776.s005.docx]

# Supplementary Material S5: Agentic Systems and Automation

This supplementary material addresses large language model capabilities that extend beyond conversational interaction, including code execution, web search, file manipulation, and multi-step workflow orchestration. These agentic capabilities fundamentally change what models can accomplish and, correspondingly, what can go wrong.

## S5.1 What Agentic Systems Are

Standard model interactions follow a simple pattern: the user provides a prompt, the model generates a response, and the user evaluates the output. Many deployed assistants already go beyond producing text, using tools such as web search and code execution as a standard part of a single response. Agentic systems extend this further, enabling models to execute code and observe results, search the web for current information, read and write files on local systems, call external APIs and services, make decisions about which tools to use and in what order, and iterate autonomously until a task is complete or a stopping condition is met. The shift is from assistant that answers questions to system that completes tasks. When a researcher asks a standard model to analyze a dataset, it can only describe what analysis it would perform. An agentic system can actually run the analysis, examine the results, adjust its approach if something fails, and return completed outputs.

Several categories of agentic tools are now accessible to researchers without requiring specialized technical expertise. Code-executing environments now integrated into major model interfaces (e.g., code interpreters and sandboxed computing environments offered by Anthropic, OpenAI, and Google) can write code, execute it, observe errors or outputs, and iterate, enabling genuine data analysis within a conversation rather than mere code generation. Web-connected assistants can retrieve current information rather than relying solely on training data, partially addressing the knowledge cutoff problem, though retrieved information still requires verification. File and document processors can read multiple documents, extract information across them, and produce synthesized outputs useful for literature processing at scale. Automation platforms that connect models to external services enable workflows such as monitoring RSS feeds for relevant papers or automatically extracting methods from preprints matching specified criteria.

## S5.2 Multi-Agent Architectures

Beyond single agents with tool access, more sophisticated architectures use multiple specialized models collaborating on complex tasks. While implementing these systems currently requires technical expertise, understanding the concept helps researchers evaluate emerging tools and anticipate where the field is heading.

Rather than asking one model to handle an entire complex task, multi-agent systems divide work among specialists. A research agent might search literature and extract relevant information. A synthesis agent might combine findings into coherent summaries. A critique agent might evaluate outputs for errors and gaps. An editor agent might refine language and structure. Each agent can be optimized for its specific role through targeted prompting or model selection; the critique agent, for instance, might be explicitly instructed to be adversarial, countering the sycophancy that affects single-model interactions.

This division of labor can reduce some failure modes inherent in single-model use. When one agent critiques another’s output, the confirmation bias that affects self-evaluation is partially mitigated. Specialized agents can apply focused verification to specific output types. The separation of concerns makes complex workflows more tractable. However, multi-agent systems also introduce coordination failures between agents, error propagation across the workflow, and increased complexity in debugging and documentation. These are not plug-and-play solutions; they require careful design and ongoing oversight.

## S5.3 Implications for Research Workflows

Agentic capabilities enable research workflows that would be impractical with standard model interactions, but the same autonomy that enables complex workflows also enables complex failures.

Systematic literature processing becomes feasible at scales that would take weeks manually. An agentic system can search databases, download papers, extract structured information, and compile results across hundreds of sources. The human role shifts from execution to verification and interpretation. Iterative data analysis benefits similarly: rather than generating code that researchers must run and debug, an agentic system can execute analysis pipelines, observe failures, adjust approaches, and iterate until producing valid outputs, dramatically reducing friction in model-assisted coding. Continuous monitoring workflows can track new publications, preprint servers, or datasets matching specified criteria, providing ongoing awareness without daily manual effort. Complex document production involving multiple steps including drafting, revision, formatting, and reference management can be partially automated, with human oversight at defined checkpoints rather than requiring continuous involvement.

While these benefits are substantial, they come with amplified risks. Opacity of intermediate steps presents a fundamental challenge: when an agent makes dozens of decisions en route to a final output, understanding how that output was produced becomes difficult. A literature synthesis might reflect choices about which papers to retrieve, which sections to emphasize, and how to resolve contradictions, none of which may be visible in the final product. Errors early in agentic workflows propagate through subsequent steps: an incorrect interpretation in step three might shape everything that follows, and the final output may show no obvious signs of the upstream problem. The more capable agentic systems become, the greater the temptation to trust their outputs without verification. When a system produces correct results most of the time, the discipline required to catch failures becomes harder to maintain. Documentation also becomes substantially more complex because recording what an agentic system did is harder than logging a simple prompt-response exchange, and complete reproducibility may require capturing tool calls, intermediate outputs, decision points, and environmental state.

## S5.4 Verification for Agentic Outputs

The verification guidance from Section 4 of the main text remains essential for agentic systems, perhaps more so given the additional complexity. Agentic outputs still require evaluation based on verifiability, domain expertise, and consequence of error, but verification must now account for multi-step processes where errors can occur at any stage.

For high-stakes outputs from agentic systems, researchers should request logs of intermediate steps and tool calls where available, verify not just final outputs but the reasoning path that produced them, maintain human checkpoints at critical decision points rather than allowing fully autonomous completion, and accept that some workflows may be too opaque for high-stakes use regardless of their convenience. The goal is maintaining meaningful oversight without negating the efficiency benefits that motivated using agentic capabilities in the first place.

## S5.5 Practical Recommendations

Researchers interested in agentic capabilities should begin with single-agent tool use before attempting complex orchestration. Code execution within a conversation is a natural starting point, providing immediate value while keeping the interaction model relatively transparent. Web search integration is similarly tractable. Building familiarity with how agents behave, where they fail, and what oversight they require before expanding scope reduces the risk of automating errors at scale.

When using agentic systems, explicitly designing human checkpoints into workflows is essential. Rather than requesting that a system process 100 papers and return a synthesis, a more prudent approach asks it to process 100 papers, show extractions from the first 10 for verification, then continue. This maintains meaningful oversight without eliminating efficiency benefits.

Agentic workflows require more extensive documentation than standard model use. Beyond the prompt and response, researchers should record what tools or capabilities were enabled, what instructions or constraints governed agent behavior, logs of tool calls and intermediate outputs where available, and which outputs received human verification versus those that did not. This documentation serves both reproducibility and debugging, since understanding what went wrong when an agentic output fails requires visibility into the full process.

Finally, honest acceptance of current limitations is warranted. Agentic capabilities are genuinely useful but genuinely limited. Current systems make mistakes that require human detection and correction. They handle routine tasks better than novel or ambiguous ones. They require more oversight than their apparent fluency suggests. They change rapidly in ways that make specific workflows fragile. The appropriate stance is pragmatic experimentation: using these capabilities where they provide value, maintaining verification practices, and expecting that both capabilities and best practices will evolve substantially.

## S5.6 Looking Forward

Agentic systems will become more capable, more accessible, and more integrated into research infrastructure. The current moment is transitional: these tools are powerful enough to be useful but immature enough to require substantial caution. While specific capabilities and interfaces will change, the principles guiding effective use are likely to remain stable: verification of outputs, documentation of processes, human oversight at high-stakes decision points, and honest assessment of when automation serves research goals versus when it introduces unacceptable risk. As capabilities expand, these principles become more important rather than less.
