# Supplementary material for: A practical risk framework for large language model use in life science research
Source: PLoS Comput Biol. 2026 Sep 16;22(9):e1014776. doi: 10.1371/journal.pcbi.1014776 (PMC13581008; doi:10.1371/journal.pcbi.1014776)
Supplement: Supplementary file 6 [file pcbi.1014776.s006.docx]

# Supplementary Material S6: Ethical Considerations

This supplementary material addresses the ethical landscape governing large language model use in research. The main text treats documentation and accountability as components of responsible practice; this supplement provides the broader ethical context researchers need before adopting model assistance in their workflows. The ethical obligations described here are not peripheral constraints but are constitutive of what responsible use means.

## S6.1 Transparency and Accountability

Transparency about large language model use is becoming a baseline expectation across scientific publishing (Nature Editorial 2023; Thorp 2023). Even when disclosure is not strictly required by a target venue, proactive transparency serves researchers’ interests: norms are forming quickly, and the reputational risk of undisclosed use being discovered substantially exceeds any awkwardness of voluntary disclosure. Researchers should check current guidelines for target venues, but should also recognize that meeting minimum requirements may not be sufficient as expectations evolve.

The verification practices described in Section 4 of the main text are ethical obligations as well as practical recommendations. If a model generates a fabricated citation that appears in a published paper, the author is responsible for including a false citation; the claim that an AI made an error is not a defense but an admission of inadequate verification. These concerns carry particular weight in biomedical research, where errors can propagate through systematic reviews and clinical guidelines into patient care. The pipeline from published finding to clinical decision creates consequences that extend beyond academic reputation, reinforcing why verification standards must match the stakes of the domain.

These obligations cannot be deferred to peer review. Reviewers cannot practically verify every citation, re-run all code, or evaluate every statistical choice in sufficient detail to catch errors that authors themselves did not check. As model-assisted writing becomes more prevalent and outputs grow more polished, the gap between surface plausibility and underlying accuracy may widen in ways that strain conventional review. The responsibility for verification rests with authors at the point of production.

## S6.2 Institutional and Funder Requirements

University and funding agency policies vary widely and continue to evolve, requiring researchers to understand requirements specific to their institutional context. Major funders including NIH and NSF are developing guidance that may impose requirements beyond those of publishers (NIH 2023; NSF 2023). Some institutions have established review processes for AI use in research, while others have yet to address the issue. The prudent approach is thorough documentation regardless of current requirements, anticipating that accountability expectations will likely increase.

Researchers should also be aware of institutional policies governing the tools themselves. Some institutions have approved specific platforms, restricted others, or established requirements for how AI tools may be procured and used. Discovering a relevant policy conflict after substantial model-assisted work has been completed creates difficult choices; understanding applicable requirements before beginning is essential.

## S6.3 Data Protection

Data protection presents concerns that researchers must address before using commercial large language model services. Most commercial APIs transmit data to external servers for processing, which may conflict with data protection requirements, IRB protocols, or data use agreements governing specific datasets. Clinical data, personally identifiable information, and data covered by specific use agreements may preclude use of external services entirely.

Even when data are not explicitly protected, researchers should consider whether transmitting unpublished findings, grant applications, or manuscript drafts to commercial services aligns with their interests and institutional expectations. Commercial data handling and retention policies vary and may permit use of submitted content for model training, though major providers have introduced options to disable this. Researchers working with sensitive data should understand the specific policies of any services they use and should consider local model deployment or institutionally approved alternatives where external data transmission is problematic.

## S6.4 Equity and Access

Equity concerns warrant attention as large language model use becomes more prevalent in research. Access to frontier models typically requires paid subscriptions, creating capability disparities between well-resourced and under-resourced researchers, institutions, and geographic regions. If model assistance provides meaningful productivity advantages in grant writing, literature synthesis, or code generation, these disparities may exacerbate existing inequalities in research output and competitiveness. These disparities also affect verification capacity: the risk framework in Section 4 recommends expert consultation for high-risk outputs outside a researcher’s domain, but access to such consultation is itself unevenly distributed, compounding the disadvantage for under-resourced researchers who may have fewer colleagues or institutional resources to draw upon.

Models also perform substantially better in English than in other languages, potentially disadvantaging researchers working primarily in other languages and reducing the accessibility of model assistance to non-English-speaking research communities. Training data reflect existing biases in what has been written and published, potentially perpetuating underrepresentation of certain topics, research traditions, and geographic perspectives. These concerns do not argue against individual researchers using available tools, but they should inform how the community develops standards, how institutions consider providing access, and how researchers with access consider supporting those without.

## S6.5 Environmental Costs

Training and operating large language models consume energy and water, and the aggregate footprint of the AI industry is significant and changing rapidly. We do not reproduce specific figures here, as current estimates date quickly; researchers who wish to weigh these costs should consult up-to-date life-cycle analyses. The practical implication is modest but real: prefer efficient, targeted use over high-volume speculative querying, in proportion to the benefit gained, which is consistent with good research practice.

## S6.6 The Evolving Landscape

The ethical landscape for large language model use in research is evolving rapidly, with norms forming simultaneously across publishers, institutions, and funding agencies. Positions that are currently aspirational best practice may become requirements within the timeframe of a research program. The most defensible posture is to document thoroughly, verify rigorously, and disclose proactively, practices that will remain appropriate regardless of how specific requirements evolve.

Researchers should also recognize that the ethical questions raised by large language model use extend beyond compliance. The scientific community has not yet resolved fundamental questions about how AI contributions should be credited, whether delegating tasks to models displaces the skill-building that early-career researchers need, whether widespread model assistance homogenizes scientific prose, or where the appropriate boundaries lie between human and machine contribution to knowledge production. Engaging with these questions thoughtfully, rather than treating them as obstacles to efficient tool use, is part of what it means to be a responsible member of the scientific community.

**References**

Nature Editorial. (2023). Tools such as ChatGPT threaten transparent science; here are our ground rules for their use. Nature, 613, 612. DOI: 10.1038/d41586-023-00191-1

NIH. (2023). The Use of Generative Artificial Intelligence Technologies is Prohibited for the NIH Peer Review Process (Notice NOT-OD-23-149). https://grants.nih.gov/grants/guide/notice-files/NOT-OD-23-149.html

NSF. (2023). Notice to the Research Community: Use of Generative Artificial Intelligence Technology in the NSF Merit Review Process. https://www.nsf.gov/news/notice-to-the-research-community-on-ai

Thorp, H.H. (2023). ChatGPT is fun, but not an author. Science, 379(6630), 313. DOI: 10.1126/science.adg7879
